# Monolayer Phosphorene–Carbon Nanotube Heterostructures for Photocatalysis: Analysis by Density Functional Theory

**DOI:** 10.1186/s11671-019-3066-z

**Published:** 2019-07-12

**Authors:** Zhaogang Zhang, Meng-Qi Cheng, Qing Chen, Hong-Yu Wu, Wangyu Hu, Ping Peng, Gui-Fang Huang, Wei-Qing Huang

**Affiliations:** 1 College of Physics Science and Engineering Technology, Yichun University, Yichun, 336000 Jiangxi China; 2 School of Physics and Electronics, Hunan University, Changsha, 410082 China; 3 School of Materials Science and Engineering, Hunan University, Changsha, 410082 China

**Keywords:** Phosphorene, Carbon nanotube, Electronic structure, Photocatalytic performance, Interfacial interaction, First-principle calculations

## Abstract

One-dimensional (1D)/2D heterostructures have attracted great attention in electronic and optoelectronic fields because of their unique geometrical structures and rich physics. Here, we systematically explore electronic structure and optical performance of single-wall carbon nanotube (CNT)/phosphorene (BP) hybrids by large-scale density functional theory (DFT) computation. The results show that the interfacial interaction between CNT and BP is a weak van der Waals (vdW) force and correlates with tube diameter of CNTs. The CNT/BP hybrids have strong optical absorption compared with that of individual BP and CNT. A diameter-dependent type I or II heterojunction in CNT/BP hybrids is observed. Moreover, CNTs can not only significantly promote photogenerated carrier transfer, but also effectively improve the photocatalytic activities of BP as a co-catalyst. These findings would enrich our understanding of BP-based 1D/2D heterostructures, providing further insight into the design of highly efficient phosphorene-based or CNT-based nanophotocatalysts.

## Background

Phosphorene (BP), a newly emerging two-dimensional (2D) layered black phosphorus [1, 2], has attracted a surge of interest for energy storage, catalysis, and sensor application [3] due to the extraordinary properties such as remarkable high hole mobility (10,000 cm^2^ V^−1^ s^−1^) [4] and widely tunable band structures (0.3–2 eV) [5, 6]. However, BP easily absorbs the small molecules including external water and oxygen in ambient conditions, resulting in its instability, which hinders its practical applications [7–10]. Recent works have demonstrated that the formation of van der Waals (vdW) heterostructures between BP and other nanomaterials can extremely improve its stability because other nanomaterials grown on the surface of BP as a contact inhibitor could prevent it from reacting with the small molecules from ambient conditions [11–17]. Chen et al. reported that the electrical performance of BN-BP heterostructure shows no degradation after exposure to ambient conditions for a whole week [11]. Yuan et al. found that the BP/MoS_2_ composites exhibit high stability and excellent photocatalytic activity (62 times higher rate of hydrogen generation than that of bare BP under visible-light irradiation) [12].

The low-dimensional carbon nanomaterials such as graphene, carbon nanotubes (CNTs), and fullerene have been widely applied due to their unique physical and chemical properties [18–20]. Various carbon nanomaterials/BP composites have been designed and synthesized owing to high stability and excellent optic-electronic properties as compared to isolated phosphorene for meeting distinct electronic and optoelectronic application [21–25]. BP is stabilized by graphene as a thin passivation layer at least several months [26]. BP/g-C_3_N_4_ hybrids show excellent and stable photocatalytic activities for H_2_ evolution and rapid degradation of RhB under visible light [24]. In particular, Chen et al. directly prepared CNT/BP 1D/2D heterostructures from red phosphorus into BP in the highly dispersed CNT matrix by a thermal-vaporization transformation method, exhibiting high stability and efficient oxygen evolution reaction (OER) activity comparable to that of commercial RuO_2_ electrocatalysts because of their unique geometrical and electronic characteristics [27]. BP sheets incorporated with CNT are produced by adding *N*-methyl-2-pyrrolidone–based BP solution into the aqueous single-walled CNT dispersion and have the improved charge transfer properties and suppressed recombination rate, and the high stability in ambient conditions [28].

To exploit the application potential of CNT/BP heterojunction as photocatalysts, the electronic structures and interfacial interaction is systematically explored by large-scale density functional theory (DFT) computations. Single-walled zigzag CNTs with different diameters varied in a wide range (0.3~20.0 nm) are employed to construct the BP/CNT heterostructures, because the electron structure of CNTs changes with the diameter [29] and will therefore influence the photoelectric properties of CNT/BP nanocomposites. More importantly, the (5,0), (7,0), (8,0), and (10,0) CNTs are semiconductors, while the (3,0), (6,0), and (9,0) CNT are metallic in nature. Therefore, the investigated CNT/BP composites are representative to elucidate the exact mechanisms of excellent photoelectric activity because the carbon nanotubes used in the experiments are usually a mixture of metallic and semiconducting tubes in nature. Here, we explicitly show that the interfacial interaction in the CNT/BP hybrid is a weak vdW interaction and related to tube diameter of CNTs. All the CNT/BP hybrids have a small band gap (< 0.8 eV) and strong optical absorption compared with that of individual BP and CNT. A diameter-dependent type I or II heterojunction in CNT/BP hybrids is observed. CNTs can effectively improve the stability of BP. These findings indicate that CNT/BP hybrids should be a good candidate as a photocatalyst, which can contribute in developing highly efficient phosphorene-based or CNT-based nanophotocatalysts.

## Methods

To construct CNT/BP heterostructures, the (1 × 1 × 1) CNTs are respectively used to represent typical ∼ 0.43 nm CNTs. The calculated supercells are composed of a (1 × 5) monolayer BP (containing 20 P atoms) and different carbon tubes with length of 4.26 Å in its axial direction. This only causes minor axial strain, leading to a 1.3% lattice mismatch. The vacuum depth is as large as 15 Å for all the hybrids to avoid artificial interaction in a supercell (4.4 × 16.5 × 28 Å^3^). All of the theoretical calculations are performed using the density functional theory (DFT) method implemented in the plane wave basis CASTEP code [30]. The Perdew−Burke−Ernzerh (PBE) type of generalized gradient approximation (GGA) exchange correlation functional [31] is chosen. Although the PBE functional may underestimate the band gaps, the calculated features and tendencies in the BP/CNT hybrids should still be qualitatively reliable [32]. The interlayer van der Waals (vdW) interaction needs to be considered by employing a semi-empirical correction scheme of Grimme’s DFT-D2 method [33]. A Morkhost-Pack mesh of k points, 5 × 8 × 1 points, is used, to sample the two-dimensional Brillouin zone for geometry optimization and for calculating the density of states (DOSs). The cutoff energy for plane waves is chosen to be 400 eV, total energy, and all forces on atoms converge to less than 10^−6^ eV and 0.01 eV/Å, respectively.

## Results and Discussion

### Geometric Structure and Formation Energy

Experimental evidences exhibit that whether the CNT is the metallic or semiconducting is closely associated with their tube diameter (D) and the helicity of the arrangement of graphitic rings in their walls [34]. The controlling diameter in the fabrication of single-walled carbon nanotube (SWNT) arrays is a crucial aspect for determining their properties and their integration into practical devices [35–37]. To clarify the effect of tube diameter on the interface interaction in the CNT/BP heterostructures, seven zigzag single-walled CNTs with different diameters ranging from 2.35 to 7.83 Å (see Table 1) are chosen.

**Table 1:** The diameter, band gap E_*g*_ of pure CNTs and the formation energy (*E*_*f*_), band gap *E*_g_^*^, optical gap *E*_*o*_, and interfacial spacing (*d*) of optimized CNT/BP composites

Hybrid	Diameter (Å)	*E*_*g*_ (eV)	*E*_*g*_^a^ (eV)	*E*_*o*_ (eV)	*E*_*f*_ (eV)	*d* (Å)	Bader charge (*e*)	
							BP	CNT
CNT(10,0)/BP	7.83	0.834	0.863	0.83	− 1.697	2.931	0.142	− 0.142
^a^CNT(9,0)/BP	7.05	0	0.438	0.44	− 1.484	2.926	0.052	− 0.052
CNT(8,0)/BP	6.27	0.555	0.375	0.37	− 1.283	2.934	0.044	− 0.044
CNT(7,0)/BP	5.48	0.409	0.315	0.30	− 0.911	2.928	0.024	− 0.024
^a^CNT(6,0)/BP	4.70	0	0.218	0.21	− 0.982	2.907	0.009	− 0.009
CNT(5,0)/BP	3.92	0.136	0.190	0.17	− 0.849	2.891	0.008	− 0.008
^a^CNT(3,0)/BP	2.35	0	0.178	0.16	− 0.593	2.804	0.004	− 0.004

Note: CNT marked with ^a^indicates that the individual CNT is metallic

**Fig. 1:**
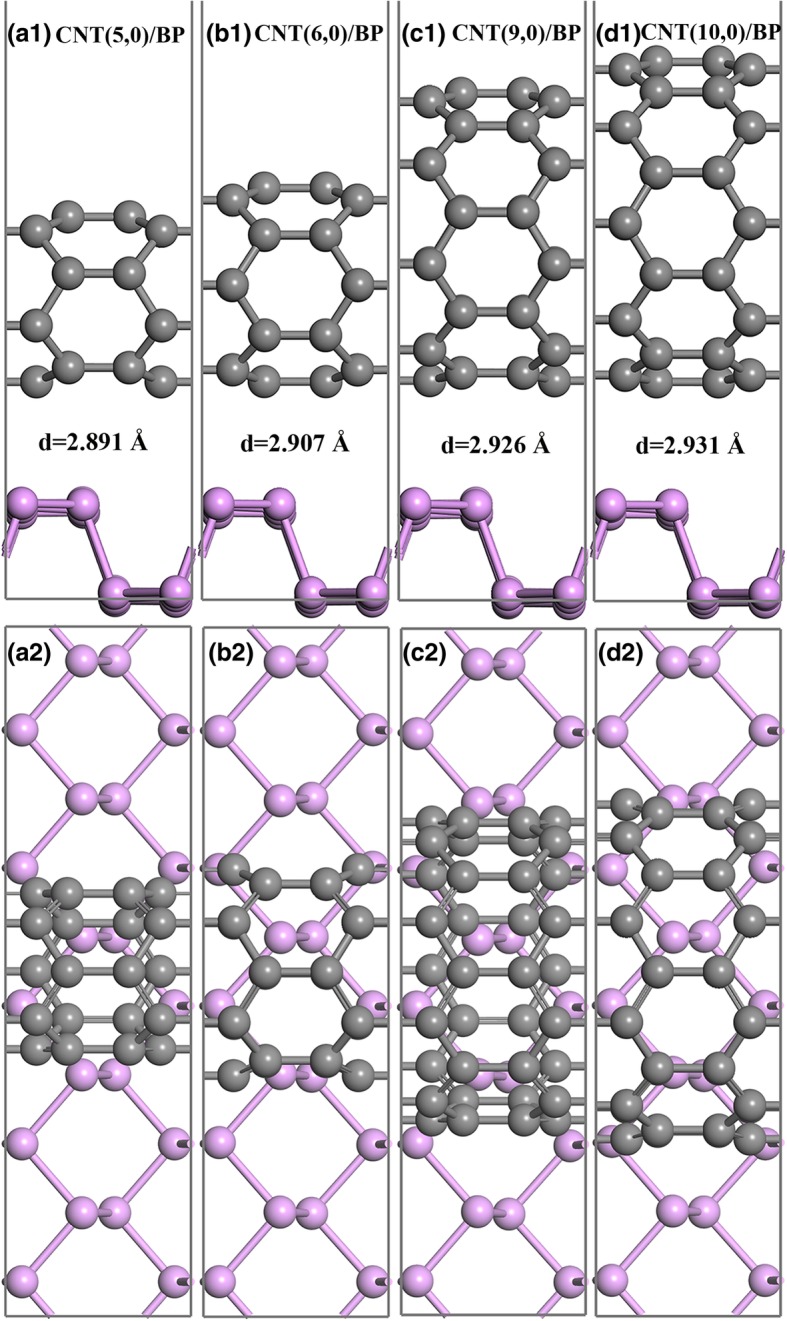
The optimized geometry for different CNTs on monolayer BP: **a1**–**d1** and **a2**–**d2** are side and top view for (5, 0), (6, 0), (9, 0), and (10, 0) CNTs, respectively. The equilibrium spacing between the nanotube wall and the top P atom layer is denoted by *d*. Gray and pink spheres represent C and P atoms, respectively

Figure 1 shows the side and top view of the optimized geometric structures for four representative CNT/BP heterostructures: (5,0) CNT/BP, (6,0) CNT/BP, (9,0) CNT/BP, and (10,0) CNT-BP hybrids, respectively. For the optimized CNT-BP hybrids, the equilibrium distances between the nanotube wall and the top P atom of monolayer BP are 2.80~2.93 Å (see Table 1), which is about comparable to those between monolayer BP (or CNT) and other materials (3.49 Å for graphene/BP [22], 3.46 Å for BN/BP [22], 2.15–3.60 Å for BP/monolayer TMD [38], 2.78–3.03 Å for MoS_2_/CNT [39], 2.73–2.86 Å for CNT/Ag_3_PO_4_ [40]). Such large equilibrium distance shows that the CNTs interact with monolayer BP through a weak vdWs force. After optimization, the CNTs and monolayer BP in the hybrids are nearly unchanged, further indicating that the CNT-BP interaction is indeed vdW rather than covalent, in consistency with the others’ results [32].

The stability of the CNT/BP hybrids can be evaluated according to their absorption energy:

1 $$ {E}_f={E}_{comb}-{E}_{CNT}-{E}_{BP} $$

where E_*comb*_, E_*CNT*_, and E_*BP*_ is the total energy of the relaxed CNT/BP, pure CNT, and monolayer BP, respectively. In accordance with the above definition, the negative *E*_*f*_ implies that the interface is stable. All the formation energy for CNT/BP hybrids are negative, almost monotone decreasing from − 0.5930 to − 1.6965 eV with increasing the tube diameter (as seen in Table 1). As a result, it is easy to conclude that these hybrids have the high thermodynamic stability and a rather strong interaction between CNT and monolayer BP. However, it is hard to distinguish that the interface coupling between the (10,0) CNT and BP is stronger than that for (3,0) CNT/BP on the basis of their formation energy. In fact, the CNT(9,0)/BP and CNT(10,0)/BP hybrids with lower formation energy would be more easily formed due to their larger contact area of the CNT with BP.

### Band Structure and Density of States

**Fig. 2:**
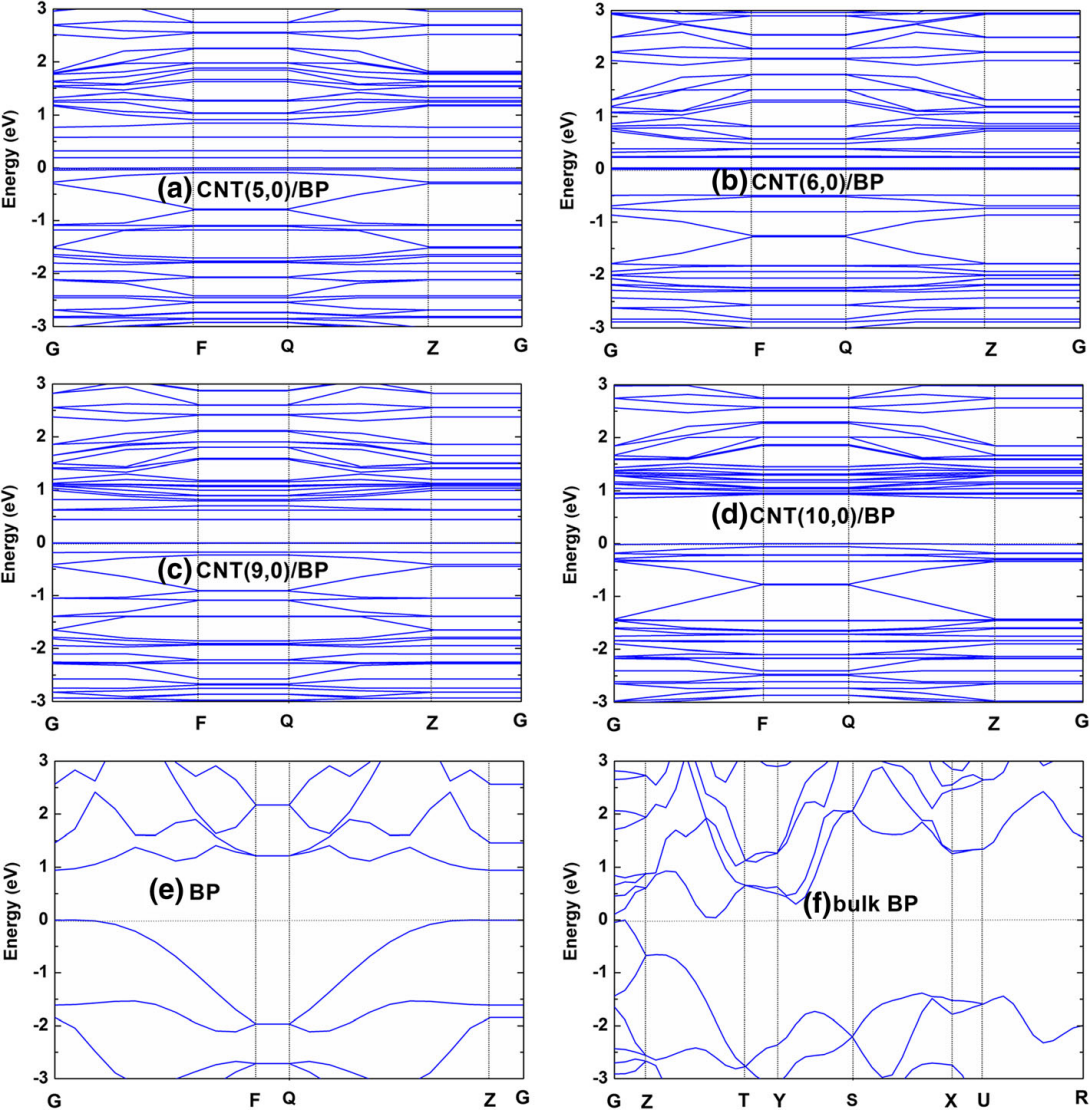
Band structures for the hybrids **a** CNT(5,0)/BP, **b** CNT(6,0)/BP, **c** CNT(9,0)/BP, **d** CNT(10,0)/BP, **e** monolayer BP, **f** bulk BP, respectively. The horizontal dashed line is Fermi level

**Fig. 3:**
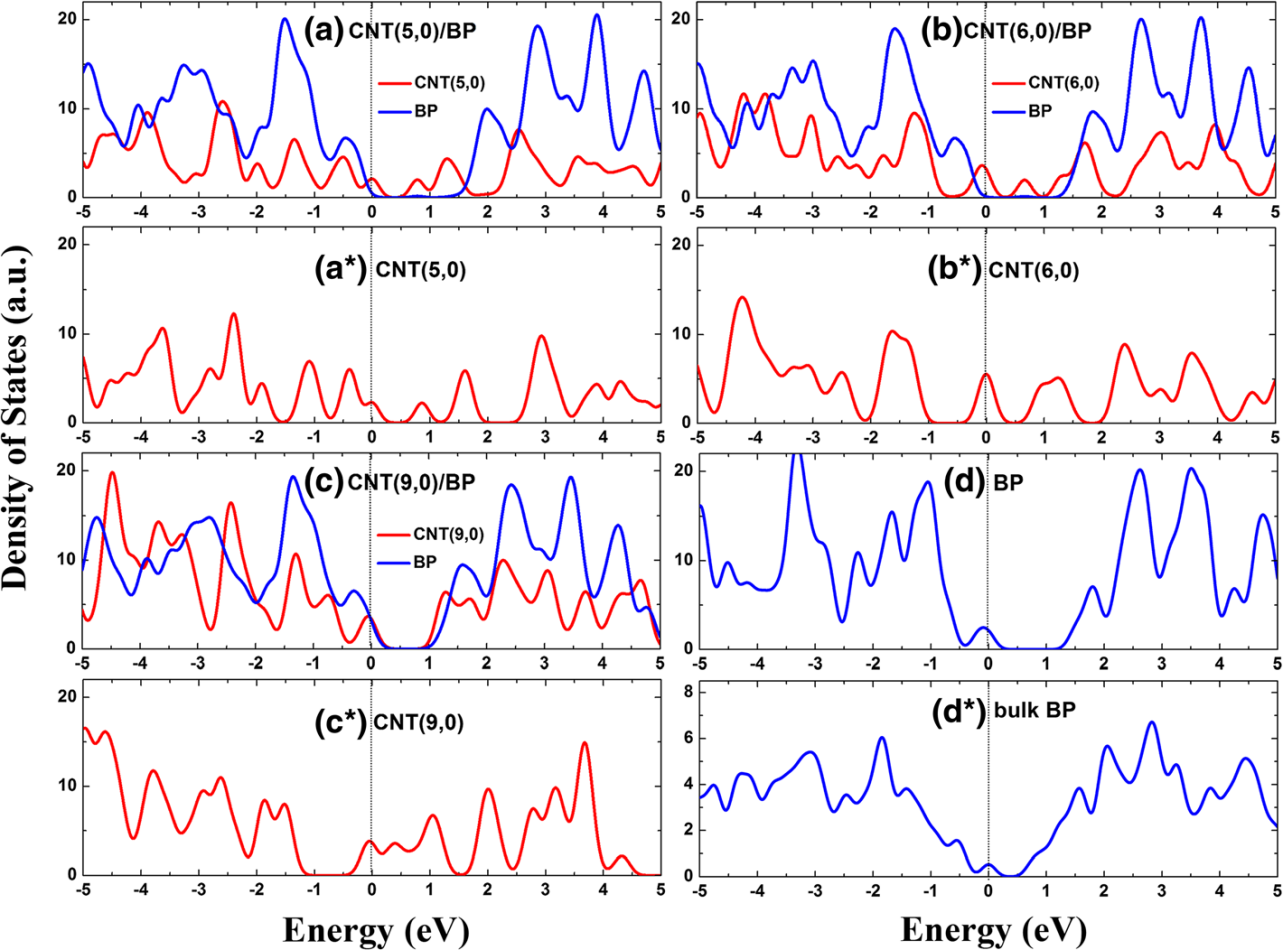
DOSs for the hybrids (**a**) CNT(5,0)/BP, (**b**) CNT(6,0)/BP, (**c**) CNT(9,0)/BP, (**d**) monolayer BP, (a*) semiconducting (5,0) CNT, (b*) metallic (6,0) CNT, (c*) metallic (9,0) CNT, and (d*) bulk BP, respectively. The Fermi level is set to zero.

In order to explore the effect of CNT on the electronic properties of monolayer BP, the band structures and density of states (DOSs) for bulk BP, monolayer BP, pure CNT, and CNT/BP hybrids are calculated (Figs. 2 and 3; Table 1). Figure 2e and f show that the valence band (VB) maximum and the conduction band (CB) minimum in our calculated bulk BP and monolayer BP are located at the G point of the Brillouin zone, which is a confirmation of the clear direct band gap (*E*_g_) semiconductor with 0.3 and 0.94 eV, consistent with previous research results [41] and their DOSs (Fig. 3d, d*). In addition, it can also be seen that the top of VB is more dispersive than the bottom of CB for BP and the hybrids, suggesting that the photogenerated holes possess smaller effective masses. The electronic characteristics in BP and the CNT/BP hybrids can promote the separation of electron–hole pairs during the reaction process and result in good photocatalytic activity.

Figure 3 shows the density of states (DOSs) of individual CNT, BP, and their hybrids. As seen in part c* of Fig. 3, (9, 0) CNT is metallic, which agrees well with the previous studies [40]. After attentively observing Fig. 3, it is easy to conclude that each component of the combined DOSs changes very little relative to those of individuals in the CNT/BP hybrids, basically maintaining the nature of their respective DOSs as isolated individuals, which is indicative of the existence of indeed weak vdW interaction at the CNT-BP interface and corresponds to large equilibrium distance between the CNT and monolayer BP in the hybrids (2.80〜2.93 Å).

The calculated band gaps of the semiconducting (5, 0), (7, 0), (8, 0), and (10, 0) CNT-BP hybrids are 0.190, 0.315, 0.375, and 0.863 eV, respectively, as listed in Table 1. Particularly, as the metallic (3,0), (6,0), and (9,0)CNTs are coupled to BP, all the metallic CNTs open a band gap due to the stress effect, similar to the previous work in CNT/MoS2 hybrids [40]. And even more interesting, the variation of band gap in the CNT/BP hybrids is in a monotonic increase with tube diameter, indicating that the influence of CNT on the electronic properties of the BP is related with the tube diameter. Therefore, it is an effective approach for the CNT/BP hybrids to tune their band gap by CNT tube diameter. In the CNT/BP hybrids, all band gaps calculated are small (< 0.9 eV, as listed in Table 1). Such band gaps have the CNT/BP hybrids absorb most of the sunlight that more photogenerated electrons are excited from the valence band (VB) to the conduction band (CB) of the heterostructures, enhancing photocatalytic performance of CNT/BP compared with monolayer BP.

**Fig. 4:**
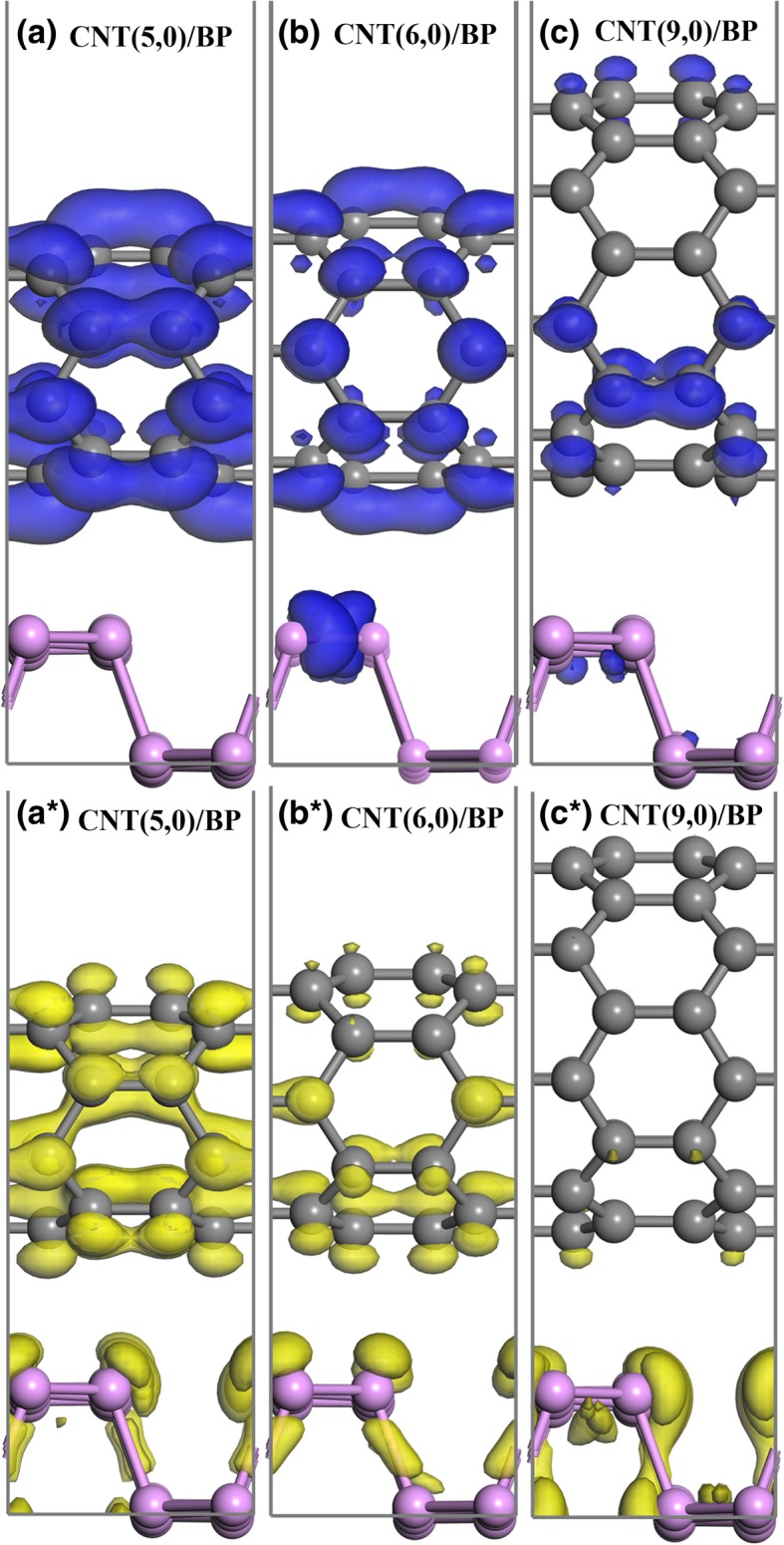
Maps of the electron and hole density distributions for LUL (**a**–**c**) and HOL (a*–c*) for the hybrid (**a**) CNT(5,0)/BP, (**b**) CNT(6,0)/BP, (**c**) CNT(9,0)/BP. The blue and yellow represent the electron and hole density distributions for LUL and HOL, respectively; the isovalue is 0.007 e/Å^3^. Herein, HOL and LUL are determined by the highest occupied and lowest unoccupied levels, respectively

Although the small band gap to capture visible light which contributes about 50% solar radiation energy plays an important role in the photocatalytic effect, it seems to be not a unique role. Actually, the effective separation of photogenerated charge carriers is also an important factor for improving photocatalytic performance [3]. As demonstrated clearly in Fig. 3, each component of the combined DOSs in the CNT/BP hybrids is mutually staggered near the Fermi level. Hence, such small band gaps in CNT/BP hybrids can be understood in a simple mechanism that the C 2p states of CNT appear in the gap of monolayer BP. Also, it is found that the near-gap electronic structure of CNT/BP hybrid varies with tube diameter. As the small CNTs (such as (5, 0) and (6, 0) tubes) are combined with monolayer BP, their energy levels are embedded in the band gap of monolayer BP (Figs. 3a, b), which can be more clearly seen from the electron density distributions of the highest occupied and lowest unoccupied levels (HOL and LUL), as demonstrated in Fig. 4. The highest occupied level (HOL) is composed of C 2p states and a small P states in CNT(6, 0)/BP, even only formed by C 2p states in CNT(5, 0)/BP, where their lowest unoccupied levels (LUL) are all composed of the C 2p orbits mixing a small P state. As a result, the CNT(5, 0)/BP and CNT(6, 0)/BP exhibited type I heterojunctions [42]. For practical purpose as photocatalysis, such band alignment is not beneficial for the separation of photogenerated electron−hole pairs but recombine readily on CNT. Consequently, the CNT might play a part of recombination centers and reduce the photocatalytic activities of CNT/BP hybrids. On the contrary, as large diameter (9, 0) CNTs are coupled to monolayer BP, their energy levels are staggered (Fig. 3c), forming a type II heterojunctions. This is also further confirmed from the two right-most columns in Fig. 4: LUL is C states and HOL is P states.

In photocatalysis, such a type II band alignment is believed to have a remarkable infIuence on the efficient separation of the photogenerated electron–hole pairs. Under light irradiation, the electrons can be directly excited from monolayer BP to CNTs and consequently result in the efficient charge separation between the two constituents. In addition, forming a type II heterostructure is an effective approach to extend the photoresponse region. As a result, a large diameter of (9, 0) CNTs is a sensitizer for monolayer BP. These results have revealed that coupling large diameter of CNTs on the monolayer BP should be a well-chosen road for achieving high photoactivity.

### Charge Density Difference and Mechanism Analysis

**Fig. 5:**
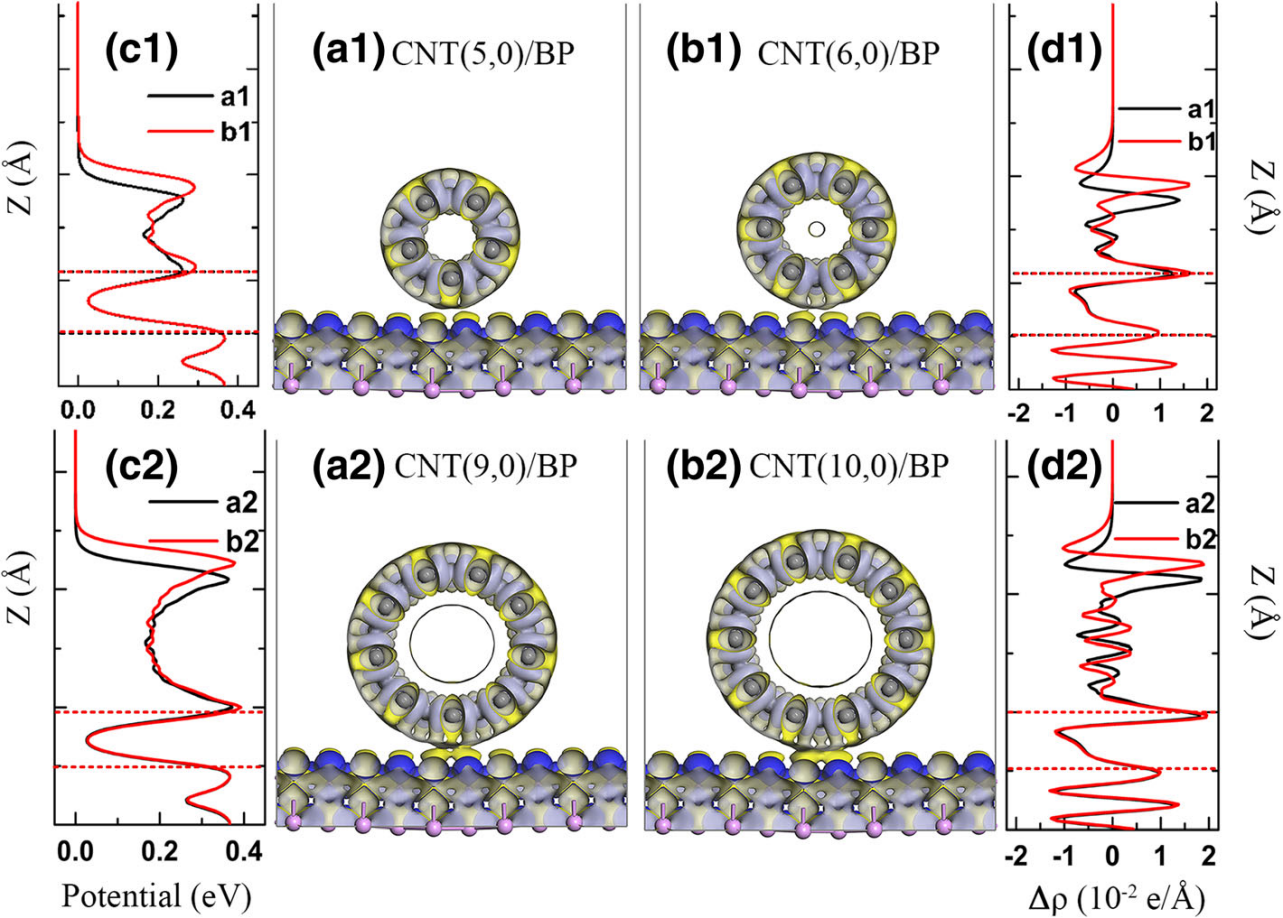
3D Charge density differences for (**a1**) CNT(5,0)/BP, (**b1**) CNT(6,0)/BP, (**a2**) CNT(9,0)/BP, and (**b2**) CNT(10,0)/BP. The yellow and blue represent charge accumulation and depletion, respectively; the isovalue is 0.0015 e/Å^3^ (**c1**). (**c2**) Profile of the planar averaged self-consistent electrostatic potential for the (**a1**) CNT(5,0)/BP, (**b1**) CNT(6,0)/BP, (**a2**) CNT(9,0)/BP, and (**b2**) CNT(10,0)/BP as a function of position in the z-direction. (**d1**), (**d2**) Profile of the planar averaged charge density difference for the (**a1**) CNT(5,0)/BP, (**b1**) CNT(6,0)/BP, (**a2**) CNT(9,0)/BP, and (**b2**) CNT(10,0)/BP as a function of position in the z-direction. The horizontal dashed line is the position of both the bottom layer of the CNT surface and the top p atom in monolayer BP

All changes of the DOSs mentioned above are originated from interfacial interaction between the involved constituents, and the interfacial interaction is highly correlated with charge transfer in heterojunctions. Actually, it can be understood in a simple mechanism based on the extent of charge transfer at the interface: the stronger coupling and the more charge transfer. Based on 3D charge density difference, the charge transfer and redistribution at the interface in these hybrids can be evaluated (as shown in Fig. 5) by the following relationship:

2 $$ \Delta  \rho ={\rho}_{CNT/ BP}-{\rho}_{BP}-{\rho}_{CNT} $$

where *ρ*_*CNT*/*BP*_, *ρ*_*BP*_, and *ρ*_*CNT*_ denote, respectively, the charge densities of the hybrids, monolayer BP, and CNT in the same configuration. In Fig. 5, the blue and yellow represent charge accumulation and depletion, respectively. Obviously, the charge redistribution is visible due to the interaction in the CNT/BP hybrid, involving all C atoms in CNT, the top p atom in BP (Fig. 5a1–b2). Moreover, a strong charge depletion (blue part in Fig. 5), is found mainly from top p atoms in BP. This indicates that CNTs are more attractive to electrons, which is helpful for enhancing the stability of monolayer BP photocatalyst.

The quantitative result of charge transfer and redistribution is plotted in Figs. 5d1 and d2 by the planar averaged charge density difference along the direction perpendicular to the monolayer BP. The horizontal dashed lines are the positions of both the bottom layer of the CNT and the top p atom of monolayer BP. The positive (negative) values indicate electron accumulation (depletion). The largest efficient electron depletion localized above the p atoms of monolayer BP is about − 1.29 × 10^−2^ e/Å^3^ in the CNT/BP hybrids, while the largest efficient electron accumulation localized at the lowest layer C atoms is about 1.41 × 10^−2^, 1.63 × 10^−2^, 1.84 × 10^−2^, and 1.96 × 10^−2^ e/Å^3^ in the CNT(5,0)/BP, CNT(6,0)/BP, CNT(9,0)/BP, and CNT(10,0)/BP hybrids. This demonstrates that the interfacial interaction between the CNT and monolayer BP gets stronger with increasing diameter of the CNT, which may be caused by the increases of the contact area between the CNT and the BP with increasing diameter of the CNT.

**Fig. 6:**
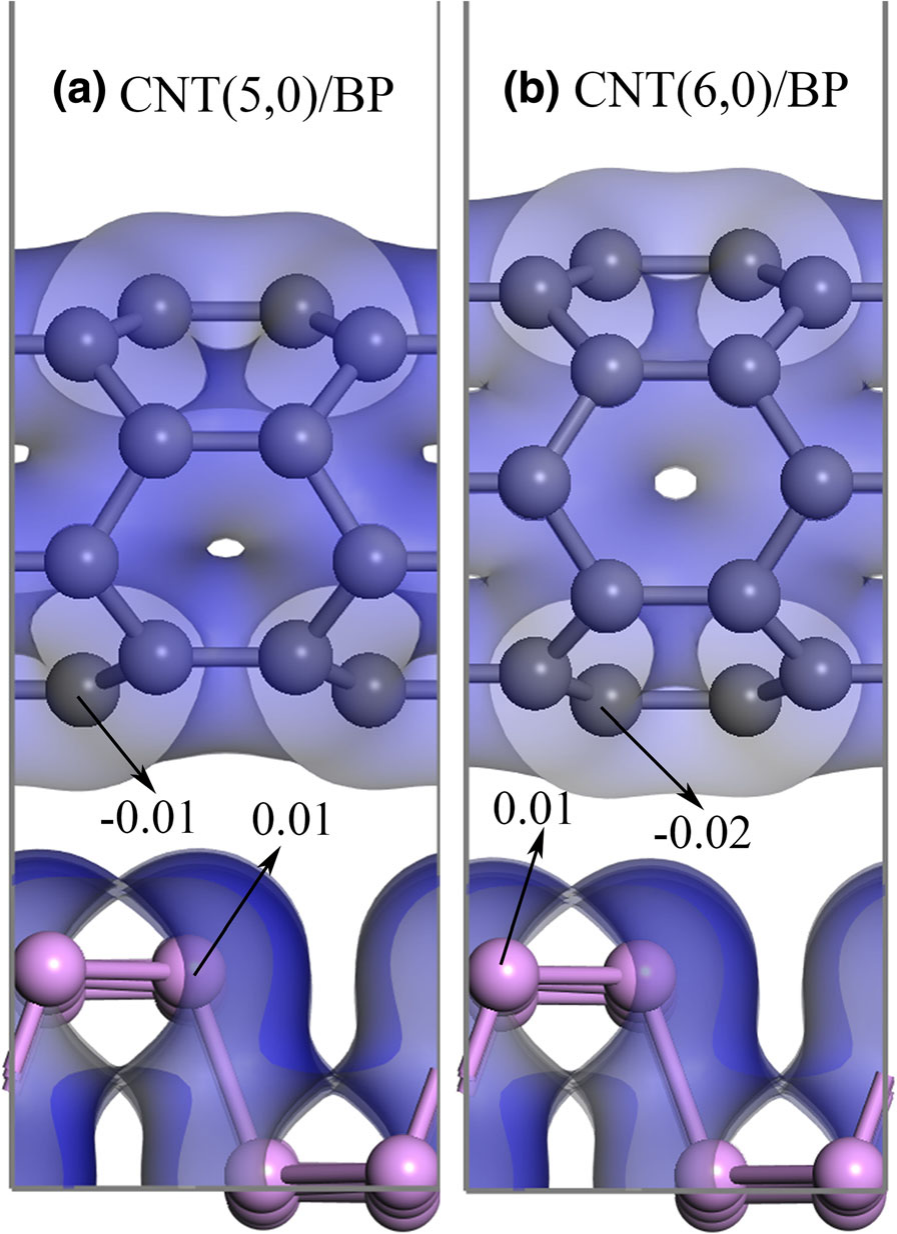
Charge distribution maps of (**a**) CNT(5,0)/BP and (**b**) CNT(6,0)/BP with an isovalue of 0.005 e/Å^3^. Gray and pink spheres represent C and P atoms

The quantitatively charge variation at the interface can also be figured out by a Mulliken population analysis of the plane wave pseudopotential calculations on the CNT, monolayer BP, and CNT/BP hybrids. Figure 6 shows the results of the Mulliken charge on C and P atoms in the CNT/BP hybrids, in which several typical values are presented. The top p atom of monolayer BP has a Mulliken charge of 0.01. The charge variation declares that the top-most P atoms of the CNT/BP hybrids would lose more electrons than those in the isolated monolayer BP (a Mulliken charge of approaching zero in pure monolayer BP).

Although the C atoms in the CNT have a Mulliken charge of approaching zero, those C atoms in the CNT/BP hybrids have different Mulliken charges because the interfacial interaction is varied. Figure 6 shows that the bottom layer C atom near monolayer BP has a Mulliken charge of − 0.01 and − 0.02 in the CNT(5,0)/BP and CNT(6,0)/BP hybrids, further depicting the interfacial interaction improvement with increasing nanotube diameter, corresponding to an increasing contact area in the CNT/BP hybrids.

The effective net charge from one constituent to another in these composites can be studied by the Bader method, as listed in Table 1. The calculated Bader charge reveals that some charge is transferred from BP to CNTs, thus leading to hole doping for the BP, which is in line with the planar averaged charge density difference. Interestingly, the amount of charge transferred in these hybrids depends upon the tube diameter. When the tube diameter becomes big (2.35–7.8 Å), the electrons transferred from BP to CNT in the CNT hybrids (Table 1) also increases (0.004–0.142), in agreement with the fact that the former has the increasing interfacial contact area in the CNT/BP hybrids.

The interfacial charge transfer will result in the variation of electrostatic potential distribution at the interface in the hybrids. Figure 5c1 and c2 display specific position in the z-direction dependence of the profile of the planar averaged self-consistent electrostatic potential for the CNT/BP hybrids. At the interface, a potential difference of ~ 0.39 eV between CNT and monolayer BP can be observed for CNT(9,0)/BP and CNT(10,0)/BP, while the average electrostatic potential difference is 0.37 eV for CNT(5,0)/BP and CNT(6,0)/BP, where there is a minor change of potential at the interface. Under light irradiation, the built-in potential at the CNT-BP interface can improve the separation and migration of photogenerated carriers in the hybrids, which would greatly enhance the photocatalytic activity and stability of the CNT/BP photocatalyst.

### Optical Properties

To assess the optical properties of monolayer BP and CNT/BP hybrids, their imaginary parts ɛ_2_ of the dielectric function are calculated from the momentum matrix elements between the occupied and unoccupied wave functions based on the Fermi golden rule within the dipole approximation by the following equation:

3 $$ {\varepsilon}_2=\frac{v{e}^2}{2\pi \mathrm{\hbar}{m}^2{\omega}^2}\int {d}^3k{\sum}_{n,n\prime }{\left|\left\langle kn\left|p\right| kn\prime \right\rangle \right|}^2f(kn)\left(1-f\left( kn^{\prime}\right)\right)\delta \left({E}_{kn}-{E}_{kn\prime }-\mathrm{\hbar}\omega \right) $$

where *ɛ*_2_, *ħɷ*, p, (| *kn*〉), and *f*(*kn*) are the imaginary part of the dielectric function, the energy of the incident photon, the momentum operator *r*(*ħ*/*i*)(*∂*/*∂x*), a crystal wave function, and Fermi function, respectively. The real part *ε*_1_(ω) of the dielectric function can be obtained from imaginary part according to Kramers–Kronig relationship. The optical absorption coefficient I(ω) can be evaluated using the following formula:

4 $$ I\left(\omega \right)=\sqrt{2}\omega {\left[\sqrt{\varepsilon \frac{2}{1}\left(\omega \right)+{\varepsilon}_2^2\left(\omega \right)}-{\varepsilon}_1\left(\omega \right)\right]}^{\raisebox{1ex}{$1$}\!\left/ \!\raisebox{-1ex}{$2$}\right.} $$

**Fig. 7:**
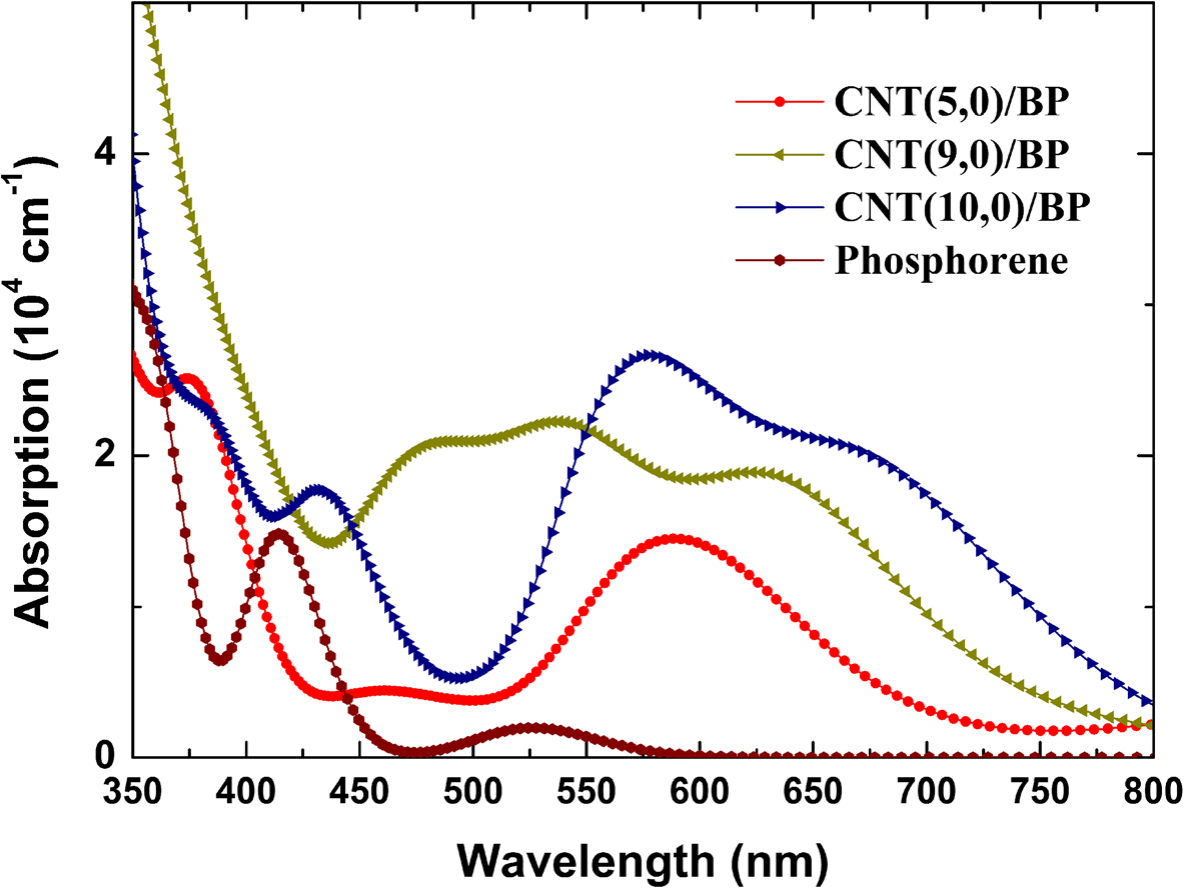
Calculated absorption spectra of the CNT/BP hybrids and pure monolayer BP

The relations above are the theoretical basis of band structure and optical properties to explain the mechanism of absorption spectral caused by electronic transition between different energy levels. Figure 7 presents the calculated UV-vis absorption spectra of monolayer BP and CNT/BP hybrids. The absorption edge of monolayer BP is located next to 0.93 eV corresponding to its intrinsic transition from the 3s to the 3p orbitals. The optical absorption edge of the CNT/BP hybrid shifts towards the longer wavelength than that of pure monolayer BP due to their decreased band gaps (see Fig. 7), as a result of the electron transition from the C 2p to P 3P states, or C 2p to C 2p states.

The strong absorption intensity is one of the most important factors for a superior photocatalyst. Compared with that of monolayer BP as illustrated in Fig. 7, the optical absorption of CNT/BP hybrids can be improved significantly in the visible-light region. It is understandable to think that the weak optical absorption of pure BP in the vis-light region is ascribed to the small values of s−p matrix elements in Eq. 3 due to the very low 3p states in the CB bottom. For the CNT/BP hybrids, C 2p- and P 3p-hybridized orbitals are predominant components at the lower part of CB and VB top (Fig. 3). The large states near the band gap of these CNT/BP hybrids correspond to the big values of *s*−*p* and *p*−*p* matrix elements in Eq. 3. Therefore, the light absorption of these CNT/BP hybrids is enhanced in the visible-light region (Fig. 7).

For the CNT/BP hybrids, the origins of the improved photocatalytic activity and stability are as follows. Firstly, the C 2p states of CNTs embedded into the band gap of BP (Fig. 3) give rise to more bound electrons taking part in the interband transitions, which not only extends the absorption range but also increases the absorption intensity compared to their individuals. Secondly, experimental results show that the BP/CNTs have a low equivalent resistance, 13 times lower than that of BP [43]. The observed excellent electrocatalytic activity and stability of BP-CNTs is much higher than that of BP, which has been attributed to much lower charge transfer resistance of BP/CNTs compared with that of BP [27]. In the CNT/BP hybrids, CNT networks with a large surface area and high conductivity play a key role of fast conductive bridge and can greatly improve the electrical conductivity of the BP catalyst. Therefore, the photogenerated charges can be shuttled freely along the conducting network of the CNT bundle under vis-light irradiation, and the photoexcited charge carriers can be effectively separated and transferred, resulting in a low carrier recombination rate and high photocatalytic activity. More importantly for the CNT(9,0)/BP hybrids, forming a type-II heterojunction band alignment (Fig. 4) makes the photoexcited electrons and holes move to different sides of heterojunction and subsequently result in an efficiently spatial separation of electron–hole pairs on before recombination [42]. Furthermore, some neutral C atoms are charged due to charge transfer in CNTs, which will become active sites from being initially catalytically inert, making the CNTs to be a highly active co-catalyst in these hybrids. Besides, the number of active sites increased significantly due to the loss of electrons during the photocatalytic process. The synergistic effects of the above factors can result in enhanced vis-light photocatalytic performance of the CNT/BP hybrids. Based on the above analysis, coupling CNT on the BP semiconductor would improve the photocatalytic activity of BP.

## Conclusions

In summary, we have investigated the potential applications of the CNT/BP composites in photocatalysis by analyzing the electronic and optical properties under the framework of DFT. Our results show that the CNT/BP hybrids have small band gap (< 0.8 eV), resulting into their strong absorption in not only vis-light region but also near-infrared spectral regions. More importantly, a type II heterojunction can effectively separate the photoexcited charge carriers in CNT(9,0)/BP hybrid and can facilitate the separation of photoexcited electrons and holes. Thus, it is reasonable to conclude that CNT/BP hybrids would be a good candidate as a photocatalyst, which can contribute to developing highly efficient phosphorene-based or CNT-based nanophotocatalysts.

## Data Availability

The datasets generated during and/or analyzed during the current study are available from the corresponding author on request.

## Abbreviations

CNT: Carbon nanotube
BP: Phosphorene
DFT: Density functional theory
vdW: van der Waals
OER: Oxygen evolution reaction
PBE: Perdew−Burke−Ernzerh
GGA: Generalized gradient approximation
DOS: Density of states
VB: Valence band
CB: Conduction band
HOL: Highest occupied levels
LUL: Lowest unoccupied levels.
